# Evolution of regular geometrical shapes in fiber lumens

**DOI:** 10.1038/s41598-017-09134-z

**Published:** 2017-08-23

**Authors:** Ngoc Lieu Le, Dooli Kim, Suzana P. Nunes

**Affiliations:** King Abdullah University of Science and Technology (KAUST), Biological and Environmental Science and Engineering Division (BESE), Thuwal, 23955-6900 Saudi Arabia

## Abstract

The geometry of polymeric hollow fibers for hemodialysis or desalination is a key factor determining their performance. Deformations are frequently observed, but they are rather random. Here we were able to exactly control the shape evolution of the internal channels or lumens of polymeric hollow fibers, leading to polygonal geometries with increasing number of sides. The elasticity of the incipient channel skin and instabilities during fiber formation are affected by the internal coagulant fluid composition and flow rate; and highly influence the polygonal shape. We propose a holistic explanation by analyzing the thermodynamic, kinetic and rheological aspects involved in the skin formation and their synergy.

## Introduction

Instabilities in polymeric fiber spinning might primarily determine the overall process economics in terms of fiber quality and fiber production rate. Particularly in the case of hollow fibers or capillary tubes the geometry of the internal channels (or lumens) is important. Instabilities or deformations can lead to mechanically weak points, which cause failure when operating at high pressures or can influence their performance^1–4^. For example, Nijdam *et al*.^1^ and Shi *et al*.^4^ reported improved water permeability of non-uniform hollow fibers due to increased surface area. Controlling production rate and fiber uniformity is very important from the industrial perspective. Understanding the conditions leading to fiber deformation and at the same time being able to control the exact shape of the internal channel (or lumen) of hollow fibers involves an intriguing combination of scientific aspects.

Hollow fibers for hemodialysis or water treatment are commonly manufactured by solution processes using an spinneret. The three primary instabilities during polymeric fiber spinning are: (i) local instabilities in polymer-lean regions in the polymer bulk flow, (ii) longitudinal instability under fiber collection and (iii) circumferential instability caused by radial/normal stresses. Understanding their fundamental mechanisms is crucial to control or overcome these instabilities. Local instability is the origin of undesirable random and disordered macrovoids or large cavities formed in fiber membranes. Macrovoid formation is influenced by interfacial tension instabilities and consequent Marangoni effect^5^, capillary convection^6, 7^, low mixing Gibbs energy between solvent and non-solvent and large chemical potential differences in the polymer solution and internal coagulant fluid medium, generating high osmotic pressure^8^ at the solution-coagulant interfaces. Macrovoid evolution and critical spinning factors, which could overcome their formation, have been extensively studied^9–11^. Longitudinal instability in fiber spinning mainly occurs when one applies a normal stress on the incipient fibers, at certain fiber collection speeds. As a consequence draw resonance and necking are observed^12^. Draw resonance is an unavoidable variation of fiber diameter, periodically occurring at the collection or take-up area, even if the fiber collection speed and the extrusion rate are constant. These small fluctuations produce oscillations in the fiber tension and further amplify the diameter fluctuations. Necking is the growth of localized indentations in the fiber cross-section. Draw resonance is known as the problem of spinnability, while necking is the cause of fiber breakage^12, 13^.

Compared to the two other instabilities above, which have been widely studied and understood, circumferential instability is less analyzed and contradictory experimental observations were obtained in various reports^14–16^. This type of spinning instability leads to fiber deformation, where its wall thickness is non-uniform and its cross-sectional shape is not circular. Depending on the origin of instability, the deformation could be random or regular. For example, the formation of vacuum condition in the internal channel of fibers produced by insufficient internal coagulant fluid supply^2^ or the partial release of accumulated back stress of the nascent fiber, due to its strongly viscoelastic properties^4^, was proven to be the reason of irregular inner contours. On the other hand, partially regular inner surfaces of fibers were observed by Bonyadi *et al*.^14^ and Yin *et al*.^17, 18^ and mass transfer and hydrodynamic, buckling, and Marangoni instabilities were proposed to explain the phenomena. Based on the proposed mechanisms, inconsistent approaches were proven to overcome these instabilities. Van’t Hoff^16^ and Pereira *et al*.^15^ suggested that using non-solvents with high coagulation strength (such as water) as internal fluid suppresses the irregularity of fiber inner shape, while Bonyadi *et al*.^14^ observed that the presence of high concentrations of non-solvents in the internal fluid induces deformed inner contours. Santoso *et al*.^2^ reported that high fiber collection speed may cause the fiber lumen deformation while Bonyadi *et al*.^14^ corrected the lumen irregularity by using a high fiber collection speed.

To understand the underlying instability that causes fiber deformation and the reasons of controversial experimental observations, for the first time we analyzed thermodynamic, rheological and kinetic aspects of the polymer system and hollow fibers preparation. Completely regular shapes of fiber inner contour were achieved and hence their corresponding interpretations are more representative.

## Results and Discussion

### Hollow fiber shape: experimental observations

**Figure 1:**
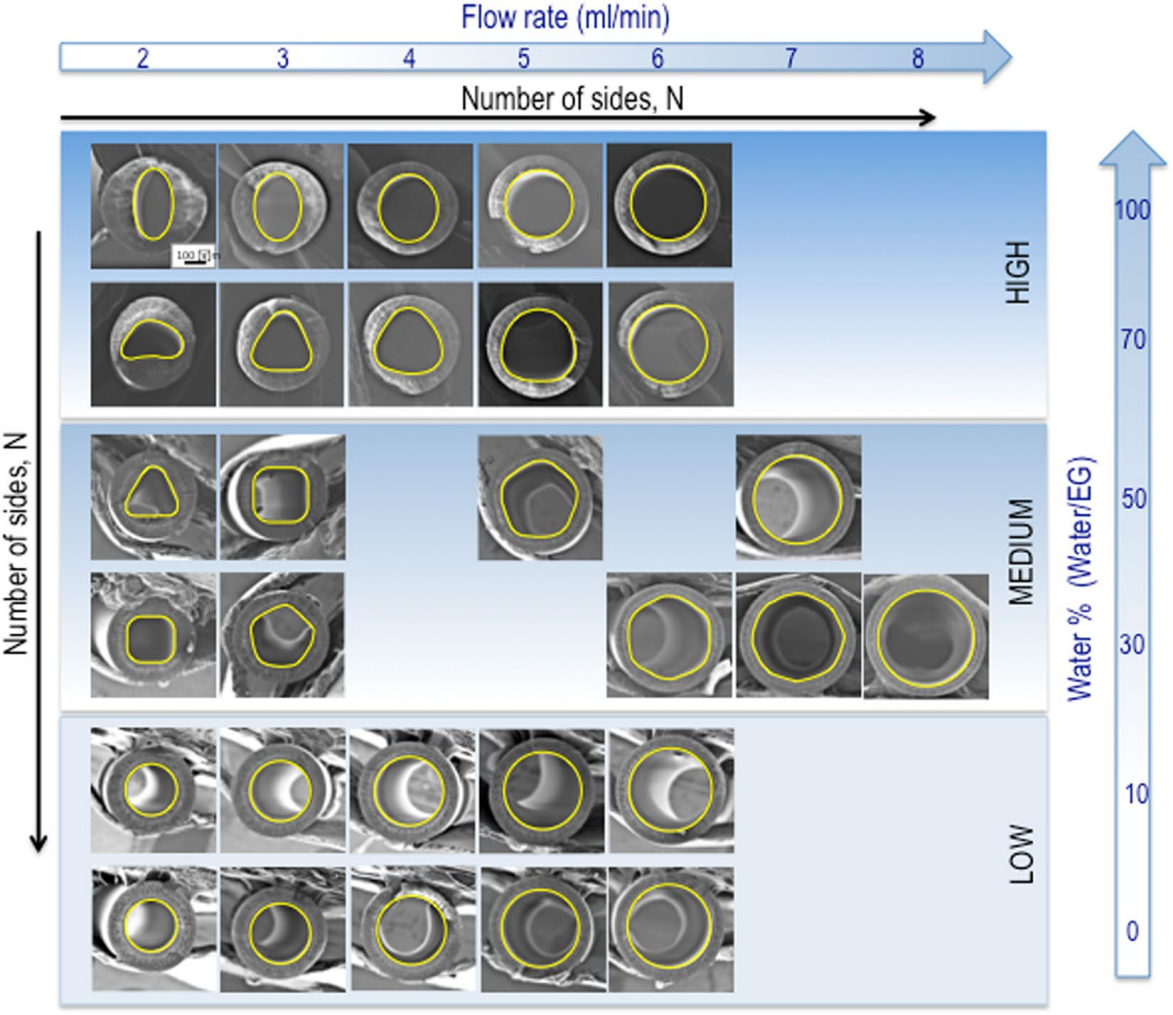
Cross-sectional morphologies of hollow fibers spun from 17/13/70 PEI/DEG//NMP polymer solution, using internal fluids with different water/EG ratios at different flow rates. The polygonal lumen shapes (with different number of sides) are highlighted in yellow.

**Figure 2:**
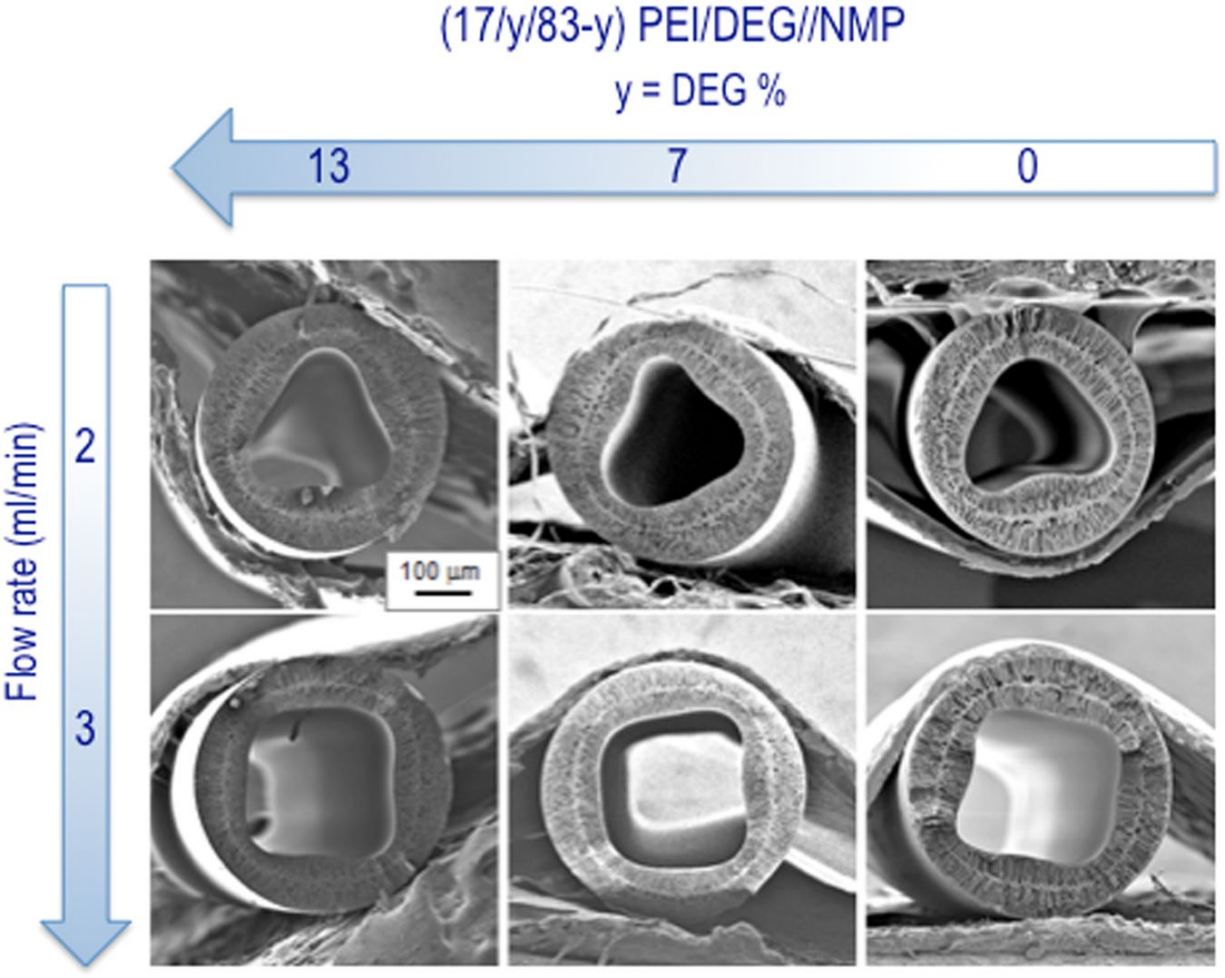
Cross-sectional morphologies of hollow fibers spun with different polymer solutions (17/13/70, 17/7/76 and 17/0/83 PEI/DEG//NMP), using 50/50 water/EG internal fluid at flow rates of 2 ml/min and 3 ml/min.

Figures 1 and 2 show the cross-sectional morphology of PEI hollow fiber membranes spun with various polymer solutions, internal fluid compositions and flow rates. The lumen assumes different shapes, not being circumferentially uniform. Different from previous reports where irregular lumen shapes were observed^2, 4, 14–18^, interestingly, they have the shapes of rounded-corner regular polygons. The number of sides, *N*, varies from 2 to 8, to a practically perfect circle in all observations.

### Effect of the internal coagulant fluid composition

Figure 1 shows the effect of internal fluid composition (i.e. water/ethylele gylcol (EG) weight ratio) and flow rates on the lumen shape evolution. Increasing the EG content in the internal fluid, going from pure water to pure EG at the constant 3 ml/min flow rate, increases *N* from 2 to 5 before the lumen shape becomes circular, keeping an identical external diameter. Our results are different from those of Santoso *et al*.’s report^2^. Although high amount of the solvent NMP in the internal fluid was previously reported to turn the deformed lumen into a circular shape, no clear evolution of regular lumen shape was observed.

### Effect of the internal fluid flow rate

Figure 1 also illustrates the effect of internal fluid flow rates on lumen shape of fibers spun with different internal fluid systems. The shape evolution, as the internal fluid flow rate increases, depends on the internal fluid composition.

*High water content* (100/0 and 70/30 water/EG): increasing the internal fluid flow rate from 2 ml/min to 6 ml/min does not rise the number of sides, but tailors their corners rounder until they reach circular shapes.

*Medium water content* (50/50 and 30/70 water/EG): *N* regularly increases with the increase of internal fluid flow rate. With the lower water/EG ratio (i.e. 30/70), the internal fluid flow rate needs to be higher to lead to a lumen with a circular shape. In particular, the shape is a square at 2 ml/min bore fluid flow rate (*Q*) and turns into circle at 8 ml/min. *N* evolution follows fine steps, going from 4 to 5, 6, 8 before a circular lumen is achieved. For the 50/50 water/EG ratio the shape is triangular at 2 ml/min and evolutes to higher *N* with relatively small internal fluid flow increase, becoming circular at 7 ml/min. By increasing the internal fluid flow rate the hollow fiber circumradius (internal and external) increases.

*Low water content* (10/90 and 0/100 water/EG): the lumen is circular, even when spun at internal fluid flow rate as low as 2 ml/min. As a result, increasing the internal fluid flow only increases its diameter without any shape changes.

For all investigated internal fluid compositions, 2 ml/min was the minimum internal fluid flow rate, for which regular lumen shape evolution could be observed for a constant polymer flow rate of 6 ml/min. Lower internal fluid flow rate (e.g. 1 ml/min) led to irregular lumen shapes, as shown in Fig. 1S. This irregularity is due to the vacuum condition in the internal channel, resulted from insufficient internal fluid supply, as reported by Santoso *et al*.^2^.

### Effect of polymer composition

Figure 2 shows the effect of the polymer composition on the hollow fiber lumen shape. The polymer concentration was constant (17 wt%), while the diethylene glycol (DEG)/ N-methyl-pyrrolidone (NMP) ratio was changed. No obvious changes in lumen shape were observed. The fibers spun with polyetherimide (PEI) solutions in three different DEG/NMP ratios had triangular shape at 2 ml/min internal fluid flow rate and square at 3 ml/min.

## Theory

### System description

In this study the air gap between the spinneret nozzle and the external coagulant bath was kept at 1 cm and the fibers were achieved with a high collection speed (12 m/min) to eliminate the effects of humidity on phase separation and minimize die-swell. When the nascent fiber reaches the coagulant bath, the polymer flow can be generally described by a 6-region model^14^. The detailed system description is provided in suporting information (Fig. 2S).

### Buckling mechanism

**Figure 3:**
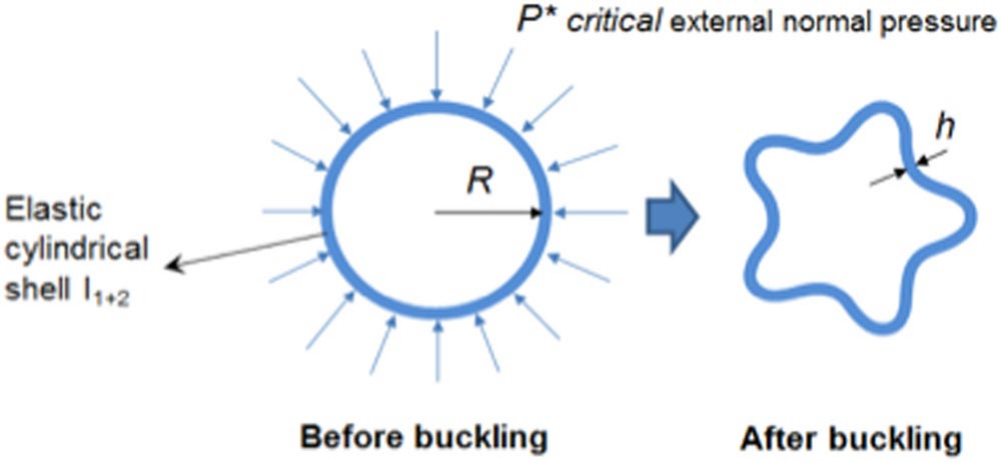
Buckling of incipient hollow fibers.

Probably the most suitable mechanism to describe the regular deformation of fiber inner contour is buckling. The elastic inner cylindrical shell I_1+2_ (Fig. 2S) could be buckled into a number of circumferential waves, as in Fig. 3, when the overall external radial pressure reaches its critical values. This pressure is the sum of the partial pressure generated by the internal fluid in lumen (*P*_1_, negative, Fig. 2S and 3S), by the fluid flow with varying density in the middle region I_3_ + O_3_ (*P*_2_, positive) and by the shrinkage of the polymer outer layer under a collection speed (*P*_3_, positive), all in the radial direction as depicted in Fig. 3S. The theory was first introduced by Levy^19^ and the equation describing the post-buckling shapes is given as below:

1 $${N}^{2}-1=12\frac{{P}^{\ast }}{E}\frac{{R}^{3}(1-{v}^{3})}{{h}^{3}}=12\frac{{P}^{\ast }}{E}m$$

where P* is the buckling critical pressure, *B* is the flexural rigidity, *R* is the radius of the circular form of the same perimeter that the fiber inner contour springs out, *E* is the modulus of elasticity, *h* is the thickness of the I_1+2_ cross-section, *ν* is the the Poisson’s ratio and *N* is the the buckling mode or the number of circumferential waves. *m* is called as dimensional lump sum. More details on the buckling theory is given in the supporting information.

The number of circumferential waves *N* depends on the external normal/radial pressure, the elasticity of the cylindrical shell I_1+2_ and the dimensions of fibers. In the other words, along with mechanical aspects, the deformation of the elastic cylindrical shell is gorverned by the combination of thermodynamic, rheological and kinetic properties of the system.

## Thermodynamic, rheological and kinetic analyses

### Thermodynamics

**Figure 4:**
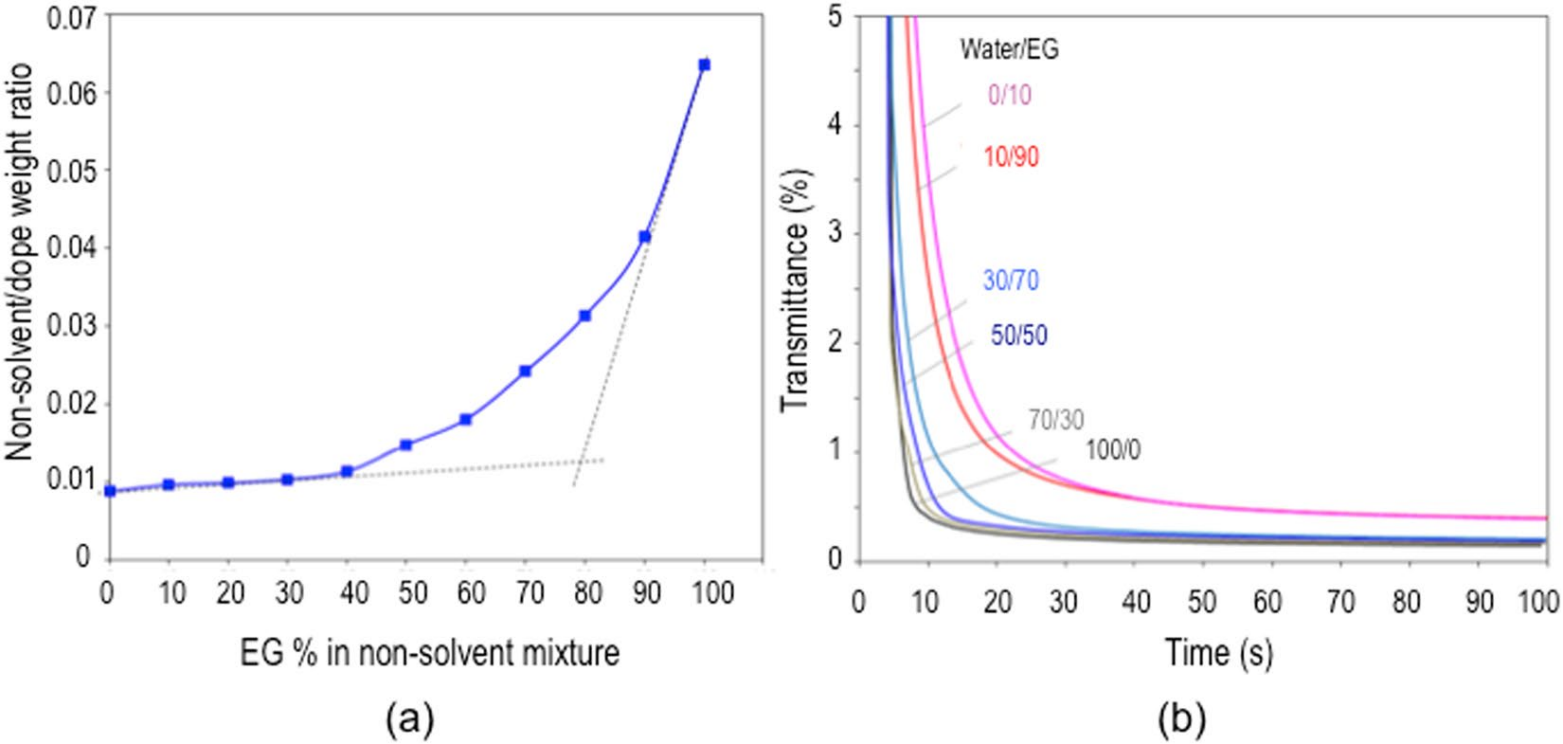
Thermodynamics and kinetics of phase separation during the fiber formation: (a) Non-solvent amount required to induce thermodynamic instability with different non-solvent compositions; (**b**) Plots of light transmittance vs time for the PEI solution in coagulants with different water/EG ratios.

Figure 4a represents the non-solvent amount required to induce thermodynamic instablility and promotes phase separation, when using different non-solvent mixtures. The miscibility between solvent, non-solvent and polymer is reflected by their solubility parameters (*δ*), which are listed in Table 1S. Compared with the common solvent NMP having a small difference in solubility parameter with PEI, water and EG are considered strong and weak non-solvents, respectively. When pure water is used as coagulant, even small amount (<1 wt%) can lead to turbidity. EG is a less strong non-solvent and hence higher amounts of water/EG mixtures are needed to lead to thermodynamic instability. When the EG percentage is ≤ 40%, the non-solvent properties of the mixture are still strong and the amounts leading to turbidity are still small. When the EG percentage is >40%, the non-solvent/polymer solution weight ratio increases gradually with the EG amount in the non-solvent mixture. It rises quickly when the EG percentage is >85%.

**Figure 5:**
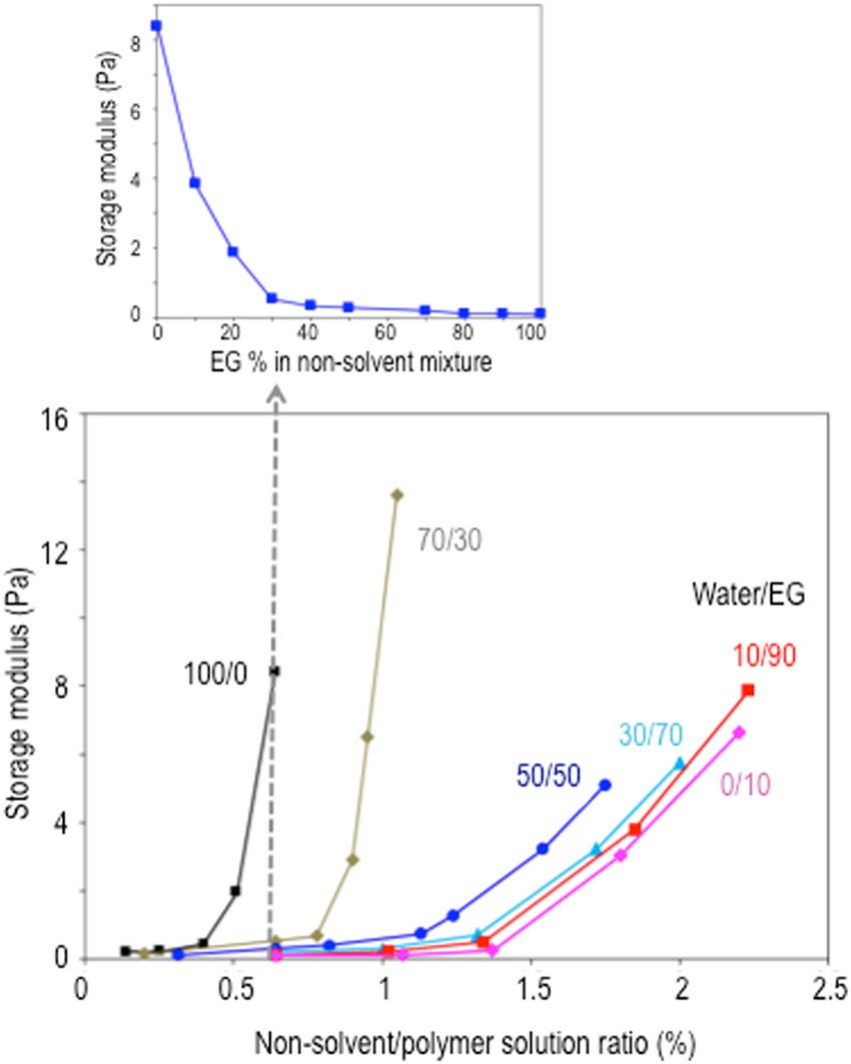
(top) Storage moduli of the PEI solutions after addition of a fixed coagulant (non-solvent) mixture amount (0.64 wt%) containing different water/EG weight ratios. This amount was selected base on 90% of the pure water amount at which the system becomes turbid.; (bottom) Storage moduli of PEI solutions as a function of the added ratios of coagulant (non-solvent) mixtures; each curve corresponds to a different coagulant composition (water/EG).

These observations are attributed to the interaction between water, EG and PEI. In pure water, water molecules interact each other via hydrogen bonding to form water clusters as illustrated in Figure 5S. When EG is added into water, it may affect the existence of water clusters and generate new forms of water-EG clusters. This change induces the site-blocking effect of EG towards water and the competition between EG and PEI in the interaction with water, which affects solvent/non-solvent exchange. Detailed explanation is given in supporting information.

### Kinetics

The phase separation kinetics, e.g. precipitation rate, influences the elasticity of the interface skin and hence the lumen deformation in hollow fiber spinning. Figure 4b depicts the precipitation kinetics of the PEI solution in the non-solvent mixture of different water/EG ratios by light transmittance measurement. Fast coagulation is observed for systems with high water fractions (100/0 or 70/30), while slow coagulation takes place when high EG fractions are used (10/90 and 0/100). This implies that the system changes from instantaneous demixing (or phase separation) to delayed demixing when more EG is added to the coagulant bath.

The precipitation/coagulation rate is strongly influenced by the non-solvent viscosity and strength, and the solvent/non-solvent miscibility. Since water has high non-solvent strength, low viscosity and high miscibility with NMP, its high fractions in the coagulant bath induces fast solvent outflow and non-solvent inflow and hence fast coagulation rate. Compared to water, EG is highly viscous (16-fold higher than water^20^) and weak in the terms of coagulant capability. Therefore, the solvent/non-solvent exchange is slow and the coagulation rate is low. Especially when a semi-solid skin is formed at the non-solvent/polymer solution interface, the high viscosity of EG retards the kinetic even more and the fiber cannot be completely solidified for a long period of immersion in the non-solvent as shown in Fig. 4b. These factors affect the elasticity of the incipient interface skin, as later discussed.

The elasticity of the interface skin is also influenced by the way that phase separation occurs. Two mechanisms govern liquid-liquid phase separation: spinodal decomposition and nucleation and growth^21, 22^. Spinodal decomposition favorably dominates in the system that abruptly falls into a two-phase condition, which may be the case of the instantaneous drop in light transmittance systems, immersed in the coagulants with high water fractions. On the other hand, slow phase separation in systems with high EG fractions induces the gradual transition from one phase to two phases and hence allows the system to stay longer in a metastable condition, promoting the nucleation and growth mechanism. Spinodal decomposition produces a denser and hence probably more elastic interface skin than nucleation and growth.

### Rheology

To determine the elasticity of the I_1+2_ region, Yin *et al*.^17^ assumed that the Young’s moduli of all layers (I_1+2_, I_3_ + O_3_ and O_1+2_) are similar and hence the Young’s modulus of the whole fiber can be representative. This assumption may not be convincing in our investigated systems because of the huge difference in porosity or structure of these layers. Instead, we then used the rheology of the non-solvent/polymer solution system to analyse the non-solvent-dependence of the elasticity of the I_1+2_ layer or the interfacial skin with the assumption that the non-solvent is well mixed with the polymer solution at the skin before it is solidified. This assumption is realistic because the skin is very thin. The storage modulus was used to investigate the capability of storing mechanical energy, which represents the elastic portion of a material.

Figure 5 shows the storage moduli of non-solvent/PEI solution sytems with different EG fractions in the non-solvent mixture. The storage modulus is high when pure water is used and quickly drops when EG is added. When the EG weight fraction is >40%, the storage moduli are similarly small. As proven in the thermodynamic analyses, water is a strong non-solvent. When the solvent/non-solvent exchange between water and NMP rapidly occurs, the interaction between solvent (now a mixture of NMP and water) and PEI decreases, allowing strong intermolecular or intramolecular interaction between PEI polymer chains and hence promoting high polymer-polymer segment friction and a high stored mechanical energy. EG is a milder non-solvent than water. When EG is present in the internal coagulant fluid, the solvent (NMP)-coagulant (water/EG) exchange leads to a milder decrease of PEI interaction with the solvent medium. The polymer-polymer segments remain plasticized and the storage modulus is small.

Figure 5 also illustrates the effect of the coagulant added to the PEI dope solution on the storage moduli. Variable amounts of up to 2.2 wt% of coagulant mixtures with different water/EG ratios were added to the polymer solution and homogenized. When the water content in the coagulant mixture is high (100 and 70%), the storage modulus abruptly increases when the non-solvent amount exceeds a certain value. On the other hand, gradual increases are observed for systems with higher EG ratios. These observations can be explained by the interaction between water, EG, NMP and PEI. Water is a much stronger coagulant for PEI than EG. When the coagulant mixture is rich in water, small amounts added are enough to drastically decrease the solvent quality and increase the polymer-polymer segment friction. EG is a weaker coagulant and larger amounts need to be added to have a comparable effect. As depicted in Figure 5S when the EG fraction is low, the water moleclues are still highly exposed and available to act as coagulant for PEI, exchanging with NMP. On the contrary, at high EG content, EG molecules entrap water in the newly formed clusters with stronger hydrogen bonds. Water is then less available to act as non-solvent, less exposed to interact with NMP and PEI.

## Overall Discussion

### Effect of internal coagulant fluid composition

As shown in Fig. 1, the change in water/EG ratio in the internal fluid does not induce a remarkable effect on fiber dimensions. Therefore, assuming a constant external normal pressure (because the fiber collection speed is kept constant), the number of circumferential waves *N* depends on the elasticity of the I_1+2_ region, as derived from Eq. (1). The rheological investigation in Fig. 5 implies that an increased EG fraction in the internal fluid lowers the elasticity and hence *n* increases as observed in Fig. 1. The circular shape of the fiber lumen, when 90% or 100% EG is used, can be explained by two aspects. First, when the elasticity reaches a certain value, the lumen polygon assumes a side number, which is high enough and has rounded corners, resembling a circle. Second, when higher EG fractions are used, an elastic interfacial skin between polymer solution and internal coagulant fluid is not formed at the early stage of phase separation (as discussed above, based on its kinetics in Fig. 5). The phase separation occurs slowly and hence no initial instability exists. As a result, the buckling phenomenon does not happen for this condition and the lumen maintains a circular shape.

### Combined effect of internal coagulant fluid composition and flow rate

When the internal fluid flow rate increases, the inner and outer diameters of fibers are expanded, while their wall thickness is narrowed as shown in Fig. 1. As a result, *R* increases and *h* decreases (in Eq. (1)) accordingly. The resultant dimensional lump sum *m* then has a remarkable increment. The change in internal fluid flow rate influences the pressure from the lumen (or internal fluid) over the internal skin and hence the overall inward external radial pressure *P*. The internal pressure on the lumen skin, which is caused by the convective flow of the internal fluid into the lumen surface outward the *r* direction, can be expressed by the pressure drop. Since the used internal fluids are incompressible and Newtonian, the pressure drop in the lumen side can be described as Eq. (2) and Figure 6S using the Hagen–Poiseuille law with assumptions that the fluid is in the laminar flow and the fluid velocity is unchanged along the *z* direction^23^.

2 $$\frac{dp}{dz}=-\frac{8{\mu }_{b}{V}_{b}}{{R}^{2}}$$

where *μ*_*b*_ and *V*_*b*_ are the viscosity and flow velocity of the internal fluid, respectively. The correlation between flow velocity *V*_*b*_ and its flow rate *Q* is decribed by Eq. (3).

3 $$Q={V}_{b}\pi {R}^{2}$$

Equation (2) indicates that the internal pressure change depends on the changes of internal fluid flow velocity *V*_*b*_ and lumen radius *R* when the internal fluid composition and hence its viscosity *μ*_*b*_ is kept constant. When the internal fluid flow rate increases, the lumen diameter is expanded at an extent that the ratio of its square value caculated from the SEM images is equal to the ratio of internal fluid flow rate as shown in Table 2S. In other words, the lumen diameter is enlarged to maintain the internal fluid velocity *V*_*b*_ constant. As a result, Eq. (2) demonstrates a decreasing trend for the internal pressure and hence the overall inward external radial pressure *P* increases with an increase in internal fluid flow rate. On the other hand, the reduced pressure drop implies a limited convective flow of the internal fluid into the polymer solution. Less internal fluid or coagulant diffusing into the polymer solution leads to lower elasticity of the interface skin. The combination of the increased *P* and *m* and the decreased *E* from Eq. (1) explains the increasing trend of circumferential waves as observed in Fig. 1.

Noticeably at a certain internal fluid flow rate, the fiber dimensions are identical, regardless of the internal fluid compositions. As a result, the changing magnitudes of *P* and *m* from one internal fluid flow rate to another are similar for different internal fluid compositions. The difference in trends in Fig. 1 is then governed by how the elasticity of the lumen skin changes. Although increasing the internal fluid flow rate leads to a lower internal fluid amount diffusing into the polymer solution and hence lower elasticity of the interface skin, the changing magnitudes depends on the internal fluid compositions. When the EG fraction is 0% or 30%, the elasticity drops quickly to very low values (less than 0.75 bar) with the added amount of coagulant mixtures. As a result, the lumen contour jumps from its original deformed shapes (ellipse or triangle) to the circular shape (Fig. 1). Transiting polygons with rounder corners are obtained when the external normal pressure *P* does not reach a critical value (P*) for the lumen skin to buckle to the next wave number with specific values of elasticity and dimensions. On the other hand, when the EG fraction is 50% or 70%, the elasticity drops gradually and hence polygons with higher numbers of sides are observed (Fig. 1). When the EG fractions are 90% or 100%, the lumen shape even at a low internal fluid flow rate of 2 ml/min is circular and hence no shape evolution is obtained (Fig. 1).

These interpretations explains the controversial experimental observations in previous reports where the internal fluids with strong coagulation strength may suppress lumen deformation^15^ or promote it^14^. The combination of thermodynamic, rheological and kinetic aspects have to be analyzed to thoroughly understand the evolution of regular lumen shapes, rather than one spinning parameter alone. In this study we observed two trends: (i) decreasing the coagulation strength of the internal fluid leads to a circular lumen shape at the internal fluid flow rate of 3 ml/min (Fig. 1) and (ii) an opposite mild tendency is obtained at internal fluid flow rates of 5 to 7 ml/min.

### Effect of different polymer solution compositions

When three different compositions were used for the polymer solution, there was probably no changes in the external normal pressure and fiber dimensions. The similarity of lumen shapes, illustrated in Fig. 2, implies that the change in elasticity of the lumen skin with polymer solution composition, in the range investigated here, is small and have no remarkable effect.

In conclusion, regular geometrical lumen shapes could be obtained by spinning hollow fibers in selected conditions. Table 1 summarizes the evolution of nascent fiber properties, its lumen shape and the correlation between spinning conditions and the different experimental or theoretical parameters discussed in this work. A clear evolution of *N*-sided rounded polygons was observed and in-depth interpreted, and the controversial experimental observations in literature were explained. Buckling mechanism was demonstrated as a suitable model to describe the regular lumen shape evolution. The lumen shape formation mechanism, evolution and hence the way to control it can be only fully understood when the thermodynamic, rheological and kinetic aspects are taken into consideration. The elasticity of the incipient interfacial skin was found as the critical factor that governs the instabilities, which dictates the evolution of the lumen shape. Rigorous mathematical simulation is needed in the future to quantitatively compute the instability leading to each *N*-shaped lumen.

**Table 1:** Evolution of nascent fiber properties and its lumen shape: correlation between spinning conditions and other parameters.

Effect on different parameters						
Change of spinning conditions	*R*	*h*	*E*	*ΔP*	*P*	*N*
Decrease of coagulation strength of the internal coagulant fluid	≈	≈	↓	≈	≈	↑
Increase of the internal fluid flow rate	↑	↓	↓	↓	↑	↑
Change of the solvent/additive ratio in the polymer solution	≈	≈	≈	≈	≈	≈
≈ no significant change; ↓ decreasing tendency; ↑ increasing tendency						

## Experimental

### Materials

Polyetherimide (PEI) Ultem^®^ 1000, provided by Sabic (Saudi Arabia), diethylene glycol (DEG, 99%, Alfa-Aesar), ethylene glycol (EG, 99.8%, Sigma-Aldrich) and N-methyl-2-pyrrolidinone (NMP, ≥ 99.5%, Merck) were used to prepare the polymer solution and internal fluids for the fabrication of hollow fibers.

### Thermodynamic analyses

The solvent/non-solvent/polymer phase diagram and the correspondent maximum amount of non-solvent added before phase separation takes place were estimated by cloud point tests, the concentration at which the turbidity of the system is first observed.

### Rheological analyses

The rheology of the non-solvent/polymer solution systems was analyzed using a AR1500ex Rheometer, TA Instruments. The storage modulus was recorded with the oscillation range of 1 to 10% and an averaged value is calculated. Each system was measured 2–3 times and their average was used.

### Phase inversion kinetics

The phase inversion kinetics of the polymer solution in various coagulant mixtures was investigated by light transmittance experiments. The polymer solution was cast on a glass plate, forming a solution layer of 1 mm and then the glass plate was immediately immersed in a cuvette, containing non-solvent. The changes in light transmittance were monitored at 600 nm (at which water, NMP and EG have no absorbance peaks) using a Cary 5000 UV-Vis-NIR, Agilent. The transmittance curves as a function of time were plotted.

### Hollow fiber spinning

PEI hollow fiber membranes were fabricated by non-solvent-induced phase-separation using a dry-jet wet spinning line. The detailed procedure can be found elsewhere^24, 25^ and spinning conditions are listed in Table 3S.

### Membrane characterizations

The cross-sectional and surface morphologies of PEI hollow fiber membranes were observed on a field emission scanning electron microscope (FESEM, Quanta 200 or Nova Nano). Before imaging, the membranes were fractured in liquid nitrogen and then coated with iridium using a sputter-coater.

## Electronic supplementary material

Supplementary information

## References

[1] Nijdam W (2005). High performance micro-engineered hollow fiber membranes by smart spinneret design. J. Membr. Sci..

[2] Santoso Y, Chung T, Wang K, Weber M (2006). The investigation of irregular inner skin morphology of hollow fiber membranes at high-speed spinning and the solutions to overcome it. J. Membr. Sci..

[3] McKelvey SA, Clausi DT, Koros WJ (1997). A guide to establishing hollow fiber macroscopic properties for membrane applications. J. Membr. Sci..

[4] Shi L (2007). Fabrication of poly (vinylidene fluoride-co-hexafluropropylene)(PVDF-HFP) asymmetric microporous hollow fiber membranes. J. Membr. Sci..

[5] Levich V, Krylov V (1969). Surface-tension-driven phenomena. Annu. Rev. Fluid Mech..

[6] Ray RJ, Krantz WB, Sani RL (1985). Linear stability theory model for finger formation in asymmetric membranes. J. Membr. Sci..

[7] Pekny MR (2002). Macrovoid pore formation in dry-cast cellulose acetate membranes: buoyancy studies. J. Membr. Sci..

[8] McKelvey SA, Koros WJ (1996). Phase separation, vitrification, and the manifestation of macrovoids in polymeric asymmetric membranes. J. Membr. Sci..

[9] Peng N, Chung TS, Wang KY (2008). Macrovoid evolution and critical factors to form macrovoid-free hollow fiber membranes. J. Membr. Sci..

[10] Widjojo N, Chung TS (2006). Thickness and air gap dependence of macrovoid evolution in phase-inversion asymmetric hollow fiber membranes. Ind. Eng. Chem. Res..

[11] Wang KY, Li DF, Chung TS, Chen SB (2004). The observation of elongation dependent macrovoid evolution in single-and dual-layer asymmetric hollow fiber membranes. Chem. Eng. Sci..

[12] Larson, R. G. Instabilities in viscoelastic flows. *Rheol. Acta* **31**, 213–263 (1992).

[13] Petrie CJ, Denn MM (1976). Instabilities in polymer processing. AIChE J..

[14] Bonyadi S, Chung TS, Krantz WB (2007). Investigation of corrugation phenomenon in the inner contour of hollow fibers during the non-solvent induced phase-separation process. J. Membr. Sci..

[15] Pereira C, Nobrega R, Borges C (2000). Spinning process variables and polymer solution effects in the die-swell phenomenon during hollow fiber membranes formation. Braz. J. Chem. Eng..

[16] Van’t Hoff, J. *Wet spinning of polyethersulfone gas separation membranes* PhD thesis, Universiteit Twente (1988).

[17] Yin J, Coutris N, Huang Y (2012). Experimental investigation of aligned groove formation on the inner surface of polyacrylonitrile hollow fiber membrane. J. Membr. Sci..

[18] Yin J, Coutris N, Huang Y (2010). Role of Marangoni instability in fabrication of axially and internally grooved hollow fiber membranes. Langmuir.

[19] Lévy, M. Memoire sur un nouveau cas integrable du probleme de l’elastique et l’une des ses applications. *Journal de Mathématiques Pures et Appliquées*, 5–42 (1884).

[20] Smallwood, I. *Handbook of organic solvent properties*. (Butterworth-Heinemann, 2012).

[21] Peinemann KV, Maggioni J, Nunes S (1998). Poly (ether imide) membranes obtained from solution in cosolvent mixtures. Polymer.

[22] Nunes SP, Inoue T (1996). Evidence for spinodal decomposition and nucleation and growth mechanisms during membrane formation. J. Membr. Sci..

[23] Yoon SH, Lee S, Yeom IT (2008). Experimental verification of pressure drop models in hollow fiber membrane. J. Membr. Sci..

[24] Sukitpaneenit P, Chung TS (2009). Molecular elucidation of morphology and mechanical properties of PVDF hollow fiber membranes from aspects of phase inversion, crystallization and rheology. J. Membr. Sci..

[25] Le NL, Nunes SP (2017). Ethylene glycol as bore fluid for hollow fiber membrane preparation. J. Membr. Sci..

