# Supplementary material for: Evolution of regular geometrical shapes in fiber lumens
Source: Sci Rep. 2017 Aug 23;7:9171. doi: 10.1038/s41598-017-09134-z (PMC5569038; doi:10.1038/s41598-017-09134-z)
Supplement: Supplementary file 1 — Supplementary information [file 41598_2017_9134_MOESM1_ESM.docx]

**Evolution of regular geometrical shapes in fiber lumens**

Ngoc Lieu Le, Dooli Kim and Suzana P. Nunes^1*^

^1^King Abdullah University of Science and Technology (KAUST),

Biological and Environmental Science and Engineering Division (BESE),

Thuwal, 23955-6900, Saudi Arabia

*Corresponding authors:

Suzana Nunes

Email address: [suzana.nunes@kaust.edu.sa](mailto:suzana.nunes@kaust.edu.sa)

Tel.: +966 544700052

**Hollow fiber spinning with internal fluid flow rates lower than 1 ml/min**


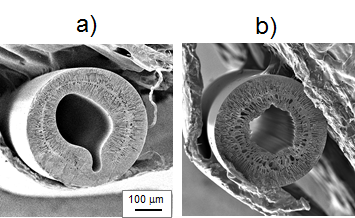


(a) (b)

**Figure 1S**. Cross-sectional morphologies of hollow fibers spun from 17/13/70 PEI/DEG//NMP polymer solution, and (a) 100/0 and (b) 50/50 water/EG as internal fluids at flow rate of 1 ml/min.

**System Description**

When the nascent fiber reaches the coagulant bath, the polymer flow can be generally described by a 6-region model^1^ as in Figure 2Sb. The top dense skin layers (I_1_ and O_1_ regions) are formed because of the rapid precipitation of polymer film surface when contacting the coagulants. The porous spongy sublayers (I_2_ and O_2_ regions) are the result of nucleation and growth of the polymer-lean phase or of spinodal decomposition. The sublayers (I_3_ and O_3_ regions) contain finger-like cavities, which are developed from diffusion/convective non-solvent flow. Since the fiber has two precipitation fronts, the fiber structure can be divided into 6 regions. When water is used as both internal and external coagulants, a virtually symmetric structure is obtained as in Figures 2Sc and 2Sd. Since the precipitation occurs rapidly in I_1_ and I_2_ (or O_1_ and O_2_), they can be approximately considered as one pseudo-homogeneous elastic cylindrical shell (I_1+2_ or O_1+2_). Since the intermediate regions I_3_ and O_3_ are overlapped and not fully solidified during spinning, they can be considered as one viscoelastic fluid (I_3_+O_3_) with varying density and rheology.


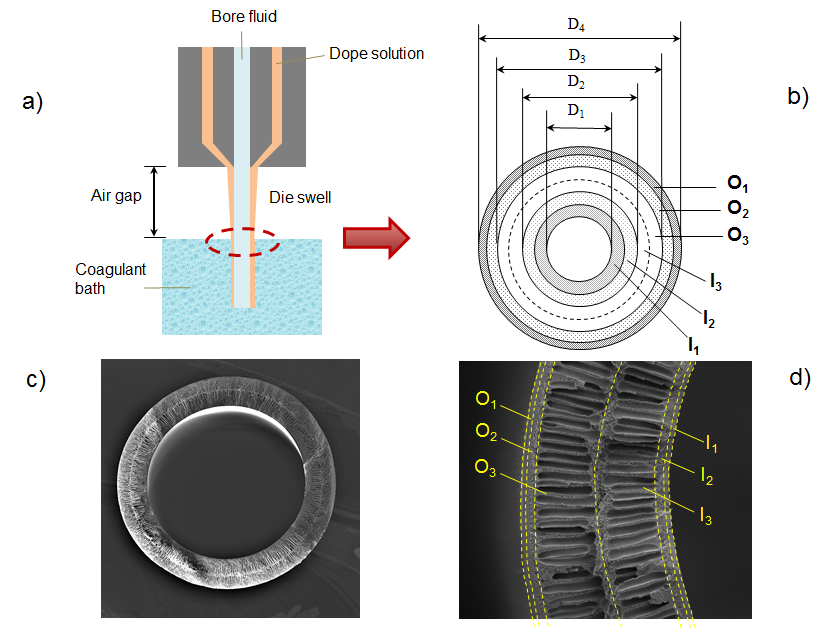


**Figure 2S**. Schematic images of (a) spinning system; (b) 6-region model of the extruded nascent fiber; and (c, d) cross-sectional morphology of the fiber spun with water as internal and external coagulants.


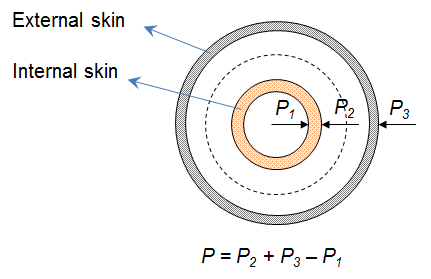


**Figure 3S**. The overall external radial pressure over the elastic inner cylindrical shell I_1+2_.

**Buckling Theory**

The theory was first introduced by Levy^2^ as Eqs. (1) and (2) and further integrated by Greenhill^3^ to describe the post-buckling shapes corresponding to each equilibrium state.

$P^{*}=\left( N^{2}-1 \right)\frac{B}{R^{3}}$ (1S)

$B=\frac{Eh^{3}}{12\left( 1-{}^{3} \right)}$ (2S)

where *P** is the buckling critical pressure, *B* is the flexural rigidity, *R* is the radius of the circular form of the same perimeter that the fiber inner contour springs out, *E* is the modulus of elasticity, *h* is the thickness of the I_1+2_ cross-section, *ν* is the the Poisson’s ratio and *N* is the the buckling mode or the number of circumferential waves. In this equation, the dimensionless parameter $k=\frac{PR^{3}}{B}$ must be ≥ 3 for the buckling to occur, where *P* is the uniform normal pressure. Its critical value of 3 corresponds to the first buckling load of the linearized theory.

Figure 4S depicts Tadjbakhsh and Odeh’s proposal^4^, where the Hamilton’s principle of minimum energy was used to derive an alternative formulation to prove the existence of buckled states for pressures greater than the first buckling pressure of the linearized theory. No buckled state is likely to exist with only one axis of symmetry. In addition, an elastic and mechanical system, for a fixed load or pressure, would be deformed into the shape of minimum energy. This non-linearized theory explains the shape difference from our observations with those in Bonyadi et al.^1^ even though the inner contours have the identical number of circumferential waves. It seems to indicate that the fiber inner shape of our study follows the linearized theory and hence Eqs. (1S) and (2S) can fully describe the observations.


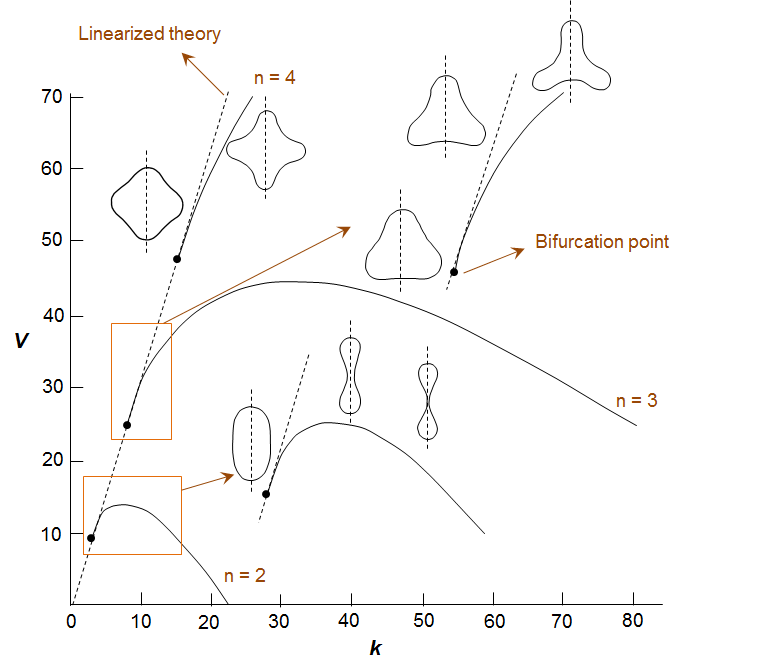


**Figure 4S**. Energy *V* as a function of the dimensionless parameter *k* including the external pressure *P* for various modes of deformation described by Tadjbakhsh and Odeh^4^.

**Effect of water-EG interaction to thermodynamics of the PEI polymer solution**

In pure water, water molecules interact each other via hydrogen bonding to form water clusters as illustrated in Figure 5Sa. These hydrogen bonds between water molecules are quickly disrupted by thermal motion and then continuously re-formed. When water is added into the PEI polymer solution, the high miscibility between water and NMP and low compatibility between water and PEI lead to demixing and solidification of the PEI rich phase.

When EG is added to water, it may affect the existence of water clusters and generate new forms of water-EG clusters. In particular, a hexatomic ring between one hydroxyl group of EG and two water molecules may be formed leading to complex water-EG clusters as depicted in the Figures 5Sb and 5Sc. In the molecular structure, these hexatomic rings were reported to strengthen hydrogen bonds between OH-containing molecules and water^5^. When the EG weight percentage is ≤ 40 %, water molecules are still highly open and mobile as depicted in Figure 5Sb and hence have high probability to directly interact and exchange with NMP molecules and efficiently induce the demixing of the system. As a result, the non-solvent amounts required to promote the solution turbidity are insignificantly different among the non-solvent mixtures with EG amount of ≤ 40 %. When the EG percentage is > 40 %, the site-blocking effect of EG may become more apparent and partially restrict the direct contact between water and NMP for solvent/non-solvent exchange to occur. The competition between EG and NMP to interact with water also plays a role. Particularly when the water/EG molar ratio is < 40/60, water molecules are virtually surrounded by EG molecules (Figure 5Sc) and hence the non-solvent function of the system becomes significantly weak.


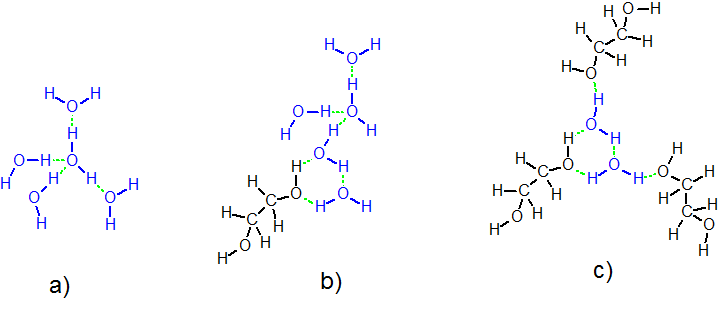


(a) (b) (c)

**Figure 5S**. Possible hydrogen bonding interaction (a) between water molecules in the liquid phase, and between water and EG molecules in (b) 5/1 and (c) 2/3 (water/EG molar ratio) solutions, corresponding to 60/40 and 16/84 water/EG weight ratio solutions, respectively.


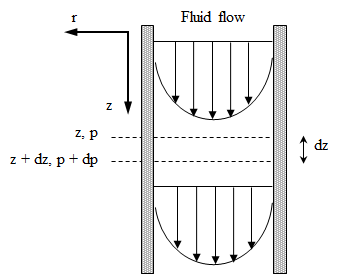


**Figure 6S**. Schematic model to describe the fluid flow in the fiber lumen. *p* is pressure, *r* and *z* are the direction.

**Table 1S**. Solubility parameters of used solvent, non-solvents and polymer^6^.

|  | **Solubility parameter (MPa^1/2^)** | | | |
| --- | --- | --- | --- | --- |
|  | **Dispersive,**  **δ_d_** | **Polar,**  **δ_p_** | **Hydrogen bonding, δ_h_** | **Total, δ_t_** |
| NMP | 18.0 | 12.3 | 7.2 | 22.9 |
| Water | 15.6 | 16.0 | 42.3 | 47.8 |
| EG | 17.0 | 11.0 | 26.0 | 32.9 |
| PEI | - | - | - | 26.3 |

**Table 2S**. Ratios of internal fluid flow rates and corresponding squares of lumen radii.

| **Ratio** | **Internal fluid flow rate (ml/min)** | | | | | |
| --- | --- | --- | --- | --- | --- | --- |
|  | 2 | 3 | 5 | 6 | 7 | 8 |
| $Q_{i}/Q_{1}$ | 1 | 1.5 | 2.5 | 3 | 3.5 | 4 |
| $R_{i}^{2}/R_{1}^{2}$ | 1 | 1.52 | 2.51 | 2.95 | 3.48 | 4.00 |

*Q_i_* and *Q_1_* are the internal fluid flow rates at 2 ml/min and other values;

*R_i_* and *R_1_* are the lumen radii at the internal fluid flow rates of 2 ml/min and values.

**Table 3S**. Spinning conditions for PEI hollow fiber membranes.

| **Spinning parameter** | **Value** | | |  |
| --- | --- | --- | --- | --- |
| Dope solution (wt.%) | 17/13/70  PEI/DEG//NMP | 17/7/76  PEI/DEG//NMP | 17/0/83  PEI/DEG//NMP |  |
| Internal fluid (wt.%) | 100/0 to 0/100 Water/EG | | |  |
| Dimensions of spinneret (mm) | 0.9/0.6 | | |  |
| External coagulant | Water | | |  |
| Temperature (◦C) | Ambient | | |  |
| Polymer solution flow rate (ml/min) | 6 | | |  |
| Internal fluid flow rate (ml/min) | 2 to 8 | | |  |
| Air gap distance (cm) | 1 | | |  |
| Fiber collection speed (m/min) | 12 | | |  |

**References**

1. Bonyadi, S., Chung, T. S. & Krantz, W. B. Investigation of corrugation phenomenon in the inner contour of hollow fibers during the non-solvent induced phase-separation process. *J. Membr. Sci.* **299**, 200-210 (2007).

2. Lévy, M. Memoire sur un nouveau cas integrable du probleme de l'elastique et l'une des ses applications. *Journal de Mathématiques Pures et Appliquées*, 5-42 (1884).

3. Greenhill, A. The Elastic Curve, under uniform normal pressure. *Mathematische Annalen* **52**, 465-500 (1899).

4. Tadjbakhsh, I. & Odeh, F. Equilibrium states of elastic rings. *Journal of Mathematical Analysis and Applications* **18**, 59-74 (1967).

5. Zhang, J. *et al.* Hydrogen bonding interactions between ethylene glycol and water: density, excess molar volume, and spectral study. *Sci. China, Ser. B: Chem.* **51**, 420-426 (2008).

6. Burke, J. Solubility parameters: theory and application. (1984).

**List of abbreviations and nomenclatures**

**Abbreviations**

DEG : diethylene glycol

EG : ethylene glycol

NMP : N-methyl-pyrrolidone

PEI : polyetherimide

**Nomenclatures**

*B* : flexural rigidity

*E* : modulus of elasticity

*h* : thickness of the I_1+2_ cross-section

*P* : uniform normal pressure

*P^*^* : buckling critical pressure

*R* : radius of the circular form of the same perimeter that the fiber inner contour springs out

*V* : storage energy

*k* : dimensionless parameter

*N* : buckling mode or the number of circumferential waves

*ν* : Poisson’s ratio

*δ* : solubility parameter
